# Pediatric Antiphospholipid Syndrome Presenting as a Massive Stroke: A Case Report

**DOI:** 10.7759/cureus.43834

**Published:** 2023-08-21

**Authors:** Fatima A Marzooq

**Affiliations:** 1 Radiology, Salmaniya Medical Complex, Manama, BHR

**Keywords:** case report, pediatric stroke, computed tomography, antiphospholipid syndrome, hemiparesis

## Abstract

Pediatric strokes are infrequent yet impactful occurrences with distinct challenges due to their unique pathophysiology and diagnostic complexities. Antiphospholipid syndrome, an autoimmune disorder characterized by antiphospholipid antibodies, can lead to prothrombotic states causing vascular occlusions. Here, we present the case of a previously healthy two-year-old girl who presented with sudden right-sided hemiparesis and altered consciousness. Comprehensive assessments and evaluations revealed a diagnosis of a massive left middle cerebral artery ischemic stroke secondary to antiphospholipid syndrome. The patient received intensive care, antiplatelet therapy, and supportive measures. Gradual improvement in neurological status and motor skills was observed during hospitalization, and the patient underwent comprehensive rehabilitation. This case emphasizes the importance of vigilance, thorough diagnostic evaluation, and tailored treatment strategies. Anticoagulation therapy plays a pivotal role, necessitating a delicate balance between thrombosis prevention and bleeding risk. The significance of multidisciplinary approaches and specialized care for pediatric stroke cases is underscored.

## Introduction

Pediatric strokes, although rare, can have profound and lasting impacts on young patients. Stroke in children presents distinct challenges due to the unique pathophysiological mechanisms and diagnostic complexities associated with this population [1]. Epidemiological studies, although limited in number due to the rarity of pediatric strokes, have revealed varying incidence rates globally [2]. Notably, studies, such as the Canadian Pediatric Ischemic Stroke Registry, a comprehensive 16-year national-based study, have shed light on the incidence, presentation, risk factors, and treatments of pediatric arterial ischemic stroke [3]. The reported incidence of childhood strokes has been documented to range from 2.5 to 13 cases per 100,000 children per year [4]. While stroke etiologies are diverse, antiphospholipid syndrome emerges as a less common yet significant cause of pediatric arterial ischemic stroke. Antiphospholipid syndrome, an autoimmune disorder characterized by the presence of antiphospholipid antibodies, contributes to a prothrombotic state, leading to vascular occlusions [5].

Pediatric antiphospholipid syndrome-associated stroke cases are particularly intriguing due to their rarity and potential for substantial neurological impairment. This case report aims to contribute to the existing medical literature by providing a detailed description of a two-year-old girl with a massive left middle cerebral artery stroke secondary to antiphospholipid syndrome. This report underscores the significance of considering antiphospholipid syndrome as a potential etiology in pediatric stroke cases, highlighting the diagnostic intricacies and therapeutic complexities that can arise in such instances.

## Case presentation

The patient, a previously healthy two-year-old girl, was brought to the emergency department by her parents with a sudden onset of right-sided hemiparesis and altered consciousness. The parents reported that the child had been in her usual state of health until the evening prior to admission when she suddenly became lethargic, was unresponsive to stimuli, and developed weakness on the right side of her body. There was no history of fever, trauma, recent illness, or family history of neurological disorders. Birth history and developmental milestones were unremarkable.

Upon admission, the child's vital signs were within normal limits. Neurological examination revealed a Glasgow Coma Scale score of 9 (E2V3M4), with decreased response to verbal and painful stimuli. The child exhibited right-sided hemiparesis with a pronounced facial droop. Pupils were equal and reactive to light, and no signs of meningeal irritation were present. Cardiovascular, respiratory, and abdominal examinations were unremarkable.

**Figure 1:**
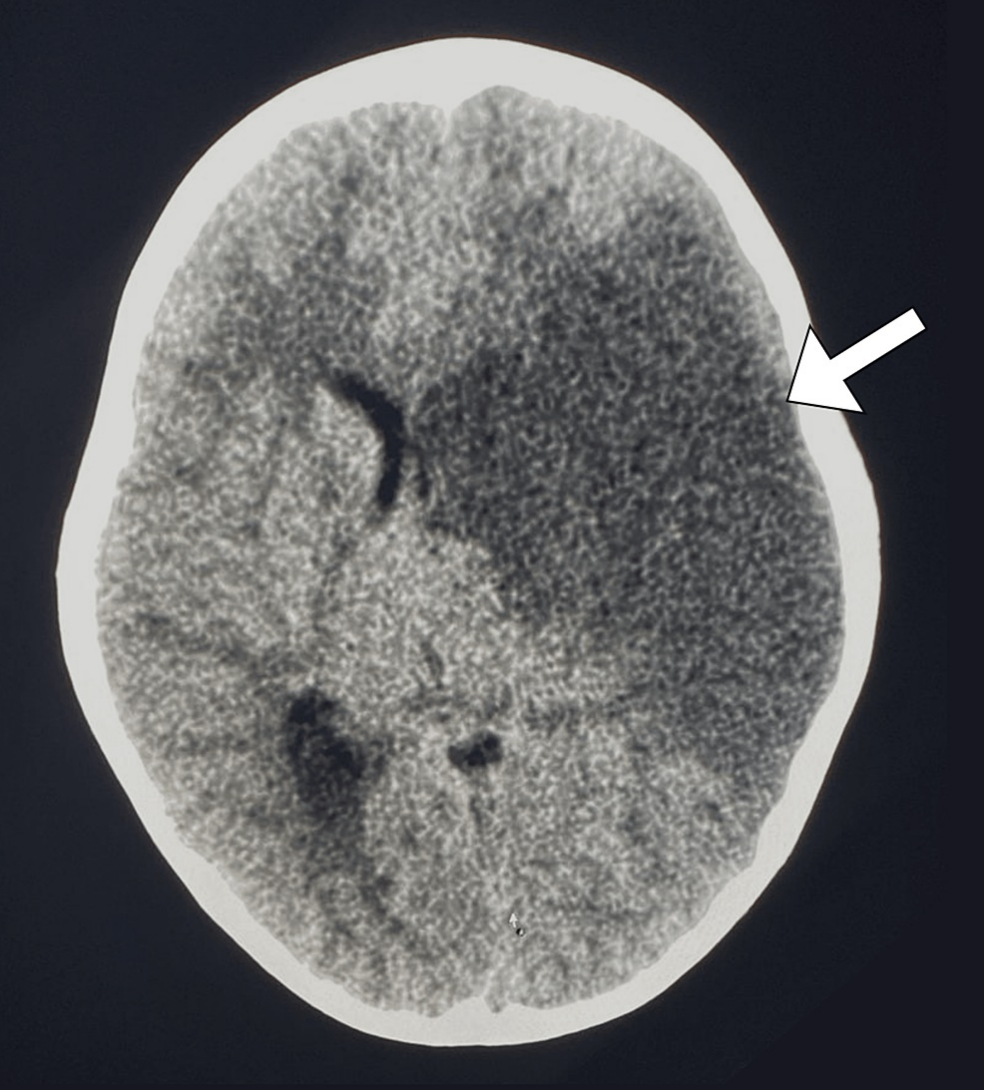
Axial CT image showing a large wedge-shaped hypodense area (arrow) in the left parietal lobe with associated loss of grey-white matter differentiation consistent with ischemic stroke CT: computed tomography

The initial differential diagnosis included acute ischemic stroke, cerebral vasculitis, metabolic disorders, and infectious causes, such as meningitis. Initial laboratory investigations, including a complete blood count, electrolytes, coagulation profile, and inflammatory markers, revealed results within normal ranges. A non-contrast computed tomography scan showed a wedge-shaped hypodensity involving the left parietal-temporal lobe, accompanied by a loss of grey-white matter differentiation. This was associated with a mass effect characterized by the effacement of sulci and midline shift to the right, along with the effacement of the ipsilateral lateral ventricle (Figure 1). Based on the clinical presentation, neuroimaging results, and exclusion of other potential causes, the patient was diagnosed with a massive left middle cerebral artery ischemic stroke.

In light of the unusual occurrence of a stroke in a two-year-old child, an extensive etiological investigation was initiated. Laboratory tests for coagulation abnormalities and specific antibodies associated with hypercoagulable states were conducted. Results revealed positive antiphospholipid antibodies, including lupus anticoagulant, anticardiolipin antibodies, and anti-β2 glycoprotein I antibodies. These findings, coupled with the clinical presentation of stroke, pointed toward a diagnosis of antiphospholipid syndrome.

The patient was promptly admitted to the pediatric intensive care unit and started on intravenous fluids and antiepileptic medications prophylactically. In addition, she was initiated on antiplatelet therapy and supportive measures to prevent further complications. Physical and occupational therapies were also initiated to aid in the recovery of motor functions.

During her hospital stay, the patient's neurological status showed gradual improvement in the level of consciousness, and she regained partial motor function in her right limbs. However, residual hemiparesis and speech difficulties persisted. The child's condition was closely monitored, and she underwent a CT scan, which showed no signs of worsening.

Upon discharge, the patient was referred to a specialized rehabilitation center for intensive therapy. Over the course of several months, she showed significant improvement in her motor skills, speech, and overall functional abilities. Regular follow-up visits with the pediatric neurology team were scheduled to monitor her progress and adjust her treatment plan accordingly.

## Discussion

The presented case of a two-year-old girl with a massive left middle cerebral artery stroke secondary to antiphospholipid syndrome serves as a noteworthy illustration of the unique challenges posed by pediatric stroke cases.

The diagnosis of antiphospholipid syndrome in a pediatric patient is relatively uncommon, emphasizing the importance of recognizing this underlying autoimmune disorder. In this case, the positive antiphospholipid antibody results, namely, lupus anticoagulant, anticardiolipin antibodies, and anti-β2 glycoprotein I antibodies, were pivotal in attributing the stroke to antiphospholipid syndrome. This aligns with studies, such as that by Sarecka-Hujar et al. [6], highlighting the association between antiphospholipid antibodies and arterial ischemic stroke in children. Moreover, the rarity of antiphospholipid syndrome in children, as opposed to adults, emphasizes the necessity for a high index of suspicion in the diagnostic process.

Antiphospholipid syndrome-associated strokes in the pediatric population often present with distinct features. In this case, the patient's sudden onset of right-sided hemiparesis and altered consciousness reflects the acute nature of pediatric strokes. The profound impact of strokes on cognitive and motor functions in pediatric patients is well-documented. The patient's gradual recovery and subsequent need for comprehensive rehabilitation mirror the outcomes reported in a study by Hart et al. [7], highlighting the importance of early intervention to optimize functional recovery.

Considering the management of antiphospholipid syndrome-associated pediatric stroke, anticoagulation therapy plays a pivotal role [5]. However, the challenge lies in balancing the benefits of preventing thrombotic events with the potential risk of bleeding complications. In line with the findings of Madison et al. [8], our patient was initiated on anticoagulation therapy, necessitating diligent monitoring of coagulation parameters. The meticulous titration of anticoagulants, as depicted in this case, reflects the nuanced management approach required in pediatric stroke cases with antiphospholipid syndrome.

The rarity of pediatric stroke events often complicates the formulation of evidence-based guidelines. The course of action in antiphospholipid syndrome-associated pediatric stroke cases is often extrapolated from adult experiences [1]. However, there is a critical need for further research and prospective studies to refine management strategies specific to the pediatric population.

## Conclusions

The presented case of a two-year-old girl with a massive left middle cerebral artery stroke secondary to antiphospholipid syndrome underscores the imperative of vigilance and a multidisciplinary approach in diagnosing and managing pediatric stroke cases. The rarity of antiphospholipid syndrome-associated strokes in the pediatric population necessitates an elevated level of suspicion, thorough antiphospholipid antibody testing, and tailored treatment strategies. As we navigate the complex landscape of pediatric stroke, this case serves as a poignant reminder of the profound impact that timely recognition and multidisciplinary interventions can have on the trajectory of a young patient's recovery.

## References

[1] Rosina S, Chighizola CB, Ravelli A, Cimaz R (2021). Pediatric antiphospholipid syndrome: from pathogenesis to clinical management. Curr Rheumatol Rep.

[2] Chiang KL, Cheng CY (2018). Epidemiology, risk factors and characteristics of pediatric stroke: a nationwide population-based study. QJM.

[3] deVeber GA, Kirton A, Booth FA (2017). Epidemiology and outcomes of arterial ischemic stroke in children: the Canadian Pediatric Ischemic Stroke Registry. Pediatr Neurol.

[4] Hollist M, Au K, Morgan L (2021). Pediatric stroke: overview and recent updates. Aging Dis.

[5] Islabão AG, Trindade VC, da Mota LM, Andrade DC, Silva CA (2022). Managing antiphospholipid syndrome in children and adolescents: current and future prospects. Paediatr Drugs.

[6] Sarecka-Hujar B, Kopyta I (2020). Antiphospholipid syndrome and its role in pediatric cerebrovascular diseases: a literature review. World J Clin Cases.

[7] Hart E, Humanitzki E, Schroeder J, Woodbury M, Coker-Bolt P, Dodds C (2022). Neuromotor rehabilitation interventions after pediatric stroke: a focused review. Semin Pediatr Neurol.

[8] Madison JA, Zuo Y, Knight JS (2020). Pediatric antiphospholipid syndrome. Eur J Rheumatol.

